# Novel pharmacological treatment options for people with eating disorders

**DOI:** 10.1192/j.eurpsy.2023.58

**Published:** 2023-07-19

**Authors:** H. Himmerich, The WFSBP Task Force on Eating Disorders

**Affiliations:** Department of Psychological Medicine, King’s College London, London, United Kingdom

## Abstract

**Abstract:**

The current scientific literature has increased our understanding of how medication could be beneficial for patients with eating disorders (EDs) on a molecular, functional, and behavioural level. Based on theoretical considerations about neurotransmitters, hormones and neural circuits, possible drug targets for the treatment of EDs may include signal molecules and receptors of the self-regulatory system such as serotonin, norepinephrine and glutamate; the hedonic system including opioids, cannabinoids and dopamine; and the hypothalamic homeostatic system including histamine, ghrelin, leptin, and insulin.

The currently approved pharmacological treatments for EDs are limited to fluoxetine for bulimia nervosa (BN) and - in some countries – lisdexamfetamine (LDX) for binge eating disorder (BED). Topiramate might be an additional option for people with BN and BED.

There are no approved pharmacological options for anorexia nervosa (AN), even though study results for olanzapine and dronabinol are promising. Psilocybin, ketamine, and metreleptin have recently been considered and tried in AN.

Case reports and studies regarding the drug treatment of the new DSM-5 EDs include the use of mirtazapine for avoidant restrictive food intake disorder (ARFID); fluoxetine for pica; and levosulpiride and baclofen for rumination disorder.

This talk is based on a comprehensive review of the scientific literature regarding the pharmacological treatment for EDs and will include a preview of the 2023 update of the World Federation of Societies of Biological Psychiatry (WFSBP) guidelines for the pharmacological treatment of eating disorders.

**Disclosure of Interest:**

None Declared

